# Expression of Genomic Instability-Related Molecules: Cyclin F, RRM2 and SPDL1 and Their Prognostic Significance in Pancreatic Adenocarcinoma

**DOI:** 10.3390/cancers13040859

**Published:** 2021-02-18

**Authors:** Anna Klimaszewska-Wiśniewska, Karolina Buchholz, Izabela Neska-Długosz, Justyna Durślewicz, Dariusz Grzanka, Jan Zabrzyński, Paulina Sopońska, Alina Grzanka, Maciej Gagat

**Affiliations:** 1 Department of Clinical Pathomorphology, Faculty of Medicine, Collegium Medicum in Bydgoszcz, Nicolaus Copernicus University in Toruń, 85-094 Bydgoszcz, Poland; karolina.buchholz@cm.umk.pl (K.B.); iznes@cm.umk.pl (I.N.-D.); justyna.durslewicz@cm.umk.pl (J.D.); d_grzanka@cm.umk.pl (D.G.); jan.zabrzynski@cm.umk.pl (J.Z.); 2 Department of Histology and Embryology, Faculty of Medicine, Collegium Medicum in Bydgoszcz, Nicolaus Copernicus University in Toruń, 85-092 Bydgoszcz, Poland; agrzanka@cm.umk.pl (A.G.); mgagat@cm.umk.pl (M.G.); 3 Department of General Orthopaedics, Musculoskeletal Oncology and Trauma Surgery, Poznan University of Medical Sciences, 60-572 Poznań, Poland; 4 Department of Obstetrics, Gynaecology and Oncology, Faculty of Medicine, Collegium Medicum in Bydgoszcz, Nicolaus Copernicus University in Toruń, 85-094 Bydgoszcz, Poland; paulina.soponska@cm.umk.pl

**Keywords:** pancreatic ductal adenocarcinoma, cyclin F, RRM2, SPDL1, genomic instability, prognostic factor

## Abstract

**Simple Summary:**

Pancreatic ductal adenocarcinoma (PDAC) is one of the worst prognostic cancers, for which clinically valuable prognostic factors and individualized biomarker-driven cancer therapies are still lacking. Recent studies have shed some light on the crucial relationship between genomic instability and PDAC progression, which can be harnessed for the cancer diagnosis, prognosis, and personalized treatment. We therefore tested the hypothesis that differences in the expression of cyclin F, RRM2, and SPLD1, i.e., proteins being implicated in maintaining genomic stability, could account for differences in clinical outcome among PDAC patients. Here, we have shown for the first time that overexpression of SPDL1 protein is a potent independent prognostic factor associated with a better survival of PDAC patients. In turn, CCNF, RRM2, and SPDL1 mRNAs are independent prognostic markers for a poor survival, both by themselves and even more in combination with each other. These biomarkers may have a potential clinical utility in the management of this deadly disease.

**Abstract:**

In the present study, we aimed to assess the selected components of cell cycle machinery, checkpoint, DNA repair, and synthesis, namely RRM2, cyclin F, and SPDL1 in pancreatic adenocarcinomas (PAC) by in-house immunohistochemistry (IHC) and bioinformatic analysis of public datasets, in terms of expression, correlation with clinicopathological parameters, and patient survival. Sixty eight patients with pancreatic ductal adenocarcinoma (PDAC) were included in our cohort study, and IHC was performed on tissue macroarrays. RNA-Seq-based transcriptome data for 177 PACs were retrieved from the Cancer Genome Atlas (TCGA). We found cyclin F, RRM2, and SPDL1 to be overexpressed at both protein and mRNA levels in tumor tissues compared to respective controls. Based on TCGA dataset, we have demonstrated that CCNF, RRM2, and SPDL1 are potent independent prognostic markers for poor overall survival, both by themselves and even more in combination with each other. Furthermore, high CCNF mRNA expression was associated with features of cancer progression. By contrast, overexpression of cyclin F or SPDL1 proteins denoted a good prognosis in PDAC patients; however, in the case of the former protein, the results did not reach statistical significance. Specifically, high levels of SPDL1 protein emerged as the most powerful independent prognostic factor associated with a better outcome. If validated, the CCNF/RRM2/SPDL1 three-gene panel developed in this study, as well as SPDL1 protein, may provide significant clinical implications for the prognosis prediction of PAC patients.

## 1. Introduction

Defects in three tightly interconnected processes throughout the cell cycle: DNA replication, DNA damage repair, and chromosome segregation are the main sources of genomic instability (GIN), being a major driving force of tumorigenesis. The clinical relevance of genomic instability is supported by its association with outcomes across various cancer types and accumulating evidence linking, e.g., chromosomal chaos (i.e., chromosomal instability, CIN) with metastasis. Indeed, GIN has been directly linked to the acquisition of malignant tumor characteristics, aneuploidy tolerance, oncogenic clonal evolution, and chemoresistance, and therefore to tumor plasticity and heterogeneity, which in turn strongly constrain the clinical benefit of personalized cancer medicine [1]. This is evident, for example, in pancreatic ductal adenocarcinoma (PDAC), which represents one of the most lethal malignancies, with an incidence almost equivalent to mortality, but still orphan of individualized biomarker-driven cancer therapy, though various genomic classifications have been reported over the years. Although PDAC may seem like a quite homogeneous disease, with recurrent mutations in four genes only (KRAS, CDKN2A, TP53, and SMAD4), it actually presents as a spectrum of distinct cancer cell subclones with different molecular and biological features, potentially accounting for varied clinical behavior. It is currently postulated that disease heterogeneity arises from the same mutational path due to ongoing genomic instability during progression. Thus far, whole-genome-wide studies in PDAC have revealed many genes that might underscore genomic instability, such as BRCA1, BRCA2, PALB2, ATM, ATR, ARID1A, CHEK1/2, RPA1, and MMR genes, with additional candidates likely to be identified going forward [2,3]. Simultaneously, mutational phenomena, such as chromothripsis and polyploidization, have been linked to unstable tumors and aggressive tumor behavior, and since PDAC exhibits a high frequency of both, this raises the possibility that they play a role in its development and progression. However, the landscape of genetic aberrations is considered insufficient to explain pervasive gene/protein expression changes, as well as alterations to cellular function, and finally the clinical heterogeneity among PDAC patients. Therefore, the search of the origin of PDAC heterogeneity has recently been focused on other mechanisms regulated at a post-genetic level. In this context, epigenetic landscape and resulting transcriptome have been used to classified PDAC tumors into specific subtypes, which more accurately correlated with therapeutic response and clinical outcome [2,4,5]. Furthermore, as it is evident that mRNA levels do not necessarily correlate with protein levels, and that proteins carry out the vast majority of cellular functions, a direct search of protein biomarkers for PDAC has also been widely conducted, but still an urgent need for new ones remains. There is now a strong belief that uncovering the mechanisms at the root cause of GIN brings the possibility to identify potential biomarkers and new therapies with more specific targets based on PDAC phenotype [2]. 

Since GIN acts as a fuel for cell-to-cell variation, targeting the specific genome instability mechanisms may provide a means to arrest tumor evolution and limit disease progression, especially in the early disease. Indeed, tumors often exhibit deregulated expression of specific components of GIN-linked processes; thus, the approaches that prevent genomic instability or take advantage of the cellular defects underlying (or caused by) it may preferentially target tumor over normal cells and are a promising area of pancreatic cancer research—toward more personalized and effective therapies [2,5]. Thus, further searching for aberrantly expressed GIN-related proteins in PDAC tissues and their reciprocal interplays may lead to identifying novel candidate drug targets and/or markers that are informative for the diagnosis and/or prognosis. 

Here, we assessed the immunohistochemical expression of the selected components of cell cycle machinery, checkpoint, and DNA repair pathways, namely the ribonucleotide reductase small subunit M2 (RRM2), cyclin F (also called FBXO1 or FBX1), and spindle apparatus coiled-coil protein 1 (Spindly; also referred to as SPDL1, CCDC99) in PDACs and normal peritumoral pancreatic tissues. We also evaluated whether the expression of candidate biomarker proteins may influence the clinicopathological factors and disease outcome. Moreover, the correlation of the combined expression of the studied proteins was also analyzed. The same was also assessed based on the gene expression data for the Cancer Genome Atlas (TCGA) cohort of pancreatic adenocarcinoma (PAC) patients, which were obtained from public sources. To the best of our knowledge, no studies have been reported on the joint expression of these proteins in PDAC samples. Of note, this is also the first study to evaluate cyclin F and SPDL1 immunoexpression in clinical samples of PDAC. Furthermore, only scarce data have been reported on the association of RRM2 protein expression with clinical outcome of pancreatic cancer patients. 

## 2. Materials and Methods

### 2.1. Patients and Tissue Material

Formalin-fixed paraffin-embedded (FFPE) specimens of 68 patients with pancreatic ductal adenocarcinoma (PDAC) who underwent a pancreatic resection at the Department of General, Hepatobiliary, and Transplant Surgery, Collegium Medicum in Bydgoszcz, Nicolaus Copernicus University in Torun (Poland), between 2009 and 2019, were retrieved from the archival collections of the Department of Clinical Pathology, Collegium Medicum in Bydgoszcz, Nicolaus Copernicus University in Torun (Poland). Data on patient clinical and pathological parameters were extracted from the electronic medical records, and the histopathological diagnoses were confirmed by the experienced pathologists. The following patient clinical and pathological data were registered if available: gender, age, pathologic T stage (pT), pathologic N stage (pN), distant metastasis (cM), grading, staging, tumor location, vascular invasion (VI), and perineural invasion (PNI). All tumors were reclassified in accordance with the standardized TNM eighth edition classification of the American Joint Committee on Cancer (AJCC) criteria. Fifty four of the 68 PDAC patients with the adjacent normal pancreatic tissue, together with another 11 specimens from normal peritumoral tissue of other PDAC patients, were obtained to be the control group (*n* = 65). The cohort includes equal number of males and females (34:34). The overall median age was 65 years, with ages ranging from 43 to 81 years. The median age at diagnosis for males was 65 (range: 43–79) and for females 64.5 (range: 44–81). The tumor was located in the head of the pancreas in 60 cases, while it was found in the pancreatic body and tail in five and three patients, respectively. Six patients were stage IA, 18 patients were stage IB, four patients were stage IIA, 20 patients were stage IIB, 12 patients were stage III, and two patients were stage IV. Five patients had well-differentiated, 55 patients had moderately differentiated, and eight patients poorly differentiated PDACs. Postsurgical survival data were available for 62 patients. The final follow-up was ended in January 2020, and median follow-up time was 1139 days. At the time of the last follow-up, 12 patients (19.35%) were still alive, whereas 50 (80.65%) patients were dead. Overall survival (OS) was defined as the time from resection until death of any case or until the last follow-up. Median OS was 393 days. Patient information and tumor characteristics are summarized in Table S1.

### 2.2. Immunohistochemistry on Tissue Macroarrays

Immunohistochemical (IHC) staining was performed on tissue macroarrays constructed from the representative tumor areas (with at least 80% of tumor cells) and the regions of histologically normal tissue that were adjacent to tumor tissue. One recipient block included five different large tissue fragments from donor paraffin blocks. For each IHC stain, a 3–4 μm paraffin section was cut from each tissue macroarray block using a manual rotary microtome (Accu-Cut, Sakura, Torrance, CA, USA). After deparaffinization in xylene and rehydration in graded ethanol, heat-induced epitope retrieval was carried out in either Dako high pH buffer (Dako; Agilent Technologies, Inc., Santa Clara, CA, USA) or Ventana high pH CC1 buffer (Roche Diagnostics/Ventana Medical Systems, Tucson, AZ, USA) in the automated PT Link system (Dako; Agilent Technologies, Inc., Santa Clara, CA, USA). Staining with antibodies for each protein was done in the automated staining apparatus (either in the Dako Autostainer (DakoCytomation, Carpinteria, CA, USA) or BenchMark^®^ ULTRA (Roche Diagnostics/Ventana Medical Systems, Tucson, AZ, USA) using either the Envision Flex Kit (Dako, Agilent Technologies, Inc., Santa Clara, CA, USA), ultraView Universal DAB Detection Kit (Roche Diagnostics/Ventana, Tucson, AZ, USA), or OptiView Universal DAB Detection Kit (Roche Diagnostics/Ventana, Tucson, AZ, USA). Blocking endogenous peroxidase activity, as well as nonspecific binding sites, was achieved by the incubation with 3% H_2_O_2_ for 10 min at room temperature (RT) and 3% bovine serum albumin (BSA) for 15 min at RT, respectively. Tissue sections were incubated with primary rabbit polyclonal anti-cyclin F antibody, rabbit polyclonal anti-RRM2 antibody, rabbit polyclonal anti-Spindly antibody, rabbit monoclonal anti-Ki-67 antibody, and mouse monoclonal anti-MSH6 antibody. Tissues sections were then counterstained with Mayer’s hematoxylin, followed by bluing reagent (except for RRM2), dehydrated in ascending ethanol, and cleared in a series of xylenes. Finally, the slides were cover-slipped in a Dako mounting medium (Agilent Technologies, Inc., Santa Clara, CA, USA). Details of antibodies and staining conditions used for IHC are collected in Table S2. Known positive control sections were used for each antibody based on the antibody datasheet and the Human Protein Atlas (http://www.proteinatlas.org (accessed on 26 August 2020)), whereas negative controls were obtained by omitting the primary antibody.

### 2.3. Immunostaining Evaluation

The IHC evaluation of protein expression was performed in the tumor tissue and non-neoplastic tissue taken from the surgical margins adjacent to the tumor, in a blinded fashion, by two independent pathologists using the light ECLIPSE E400 microscope (Nikon Instruments Europe, Amsterdam, The Netherlands).

The immunoexpression of studied proteins was analyzed at 20× original objective magnification and based on the modified Index Remmele–Stegner (IRS) scale, which gives a range of 0–12 as a product of multiplication between the percentage of positively stained cells/areas (0–4) and staining intensity (0–3). The scores for the positive immunoreactivity (PS) for RRM2 and Spindly were categorized as follows: (0) less than 10% of stained cells/area, (1) 11–20% of stained cells/area, (2) 21–50% of stained cells/area, (3) 51–80% of stained cell/area, and (4) equal or more than 81% of stained cells/area. In turn, the intensity score (IS) was defined as (0) negative, (1) low staining, (2) moderate staining, and (3) strong staining. In the case of cyclin F, the positive cell proportion score was classified as follows: (0) <5% positive cells/areas, (1) 5–24% positive cells/areas, (2) 25–49% positive cells/areas, (3) 50–74% positive cells/areas, and (4) ≥75% positive cells/areas, whereas the staining intensity was measured using the same numerical scale as mentioned above. Ki-67 proliferation index was determined by assessing the percentage of positively stained cancer cell nuclei in 1000 neoplastic cells within a hotspot area. For IHC scoring of MSH6 expression, minimal to low staining was graded 0, moderate staining 1, and high staining 2. There were no cases with complete absence of nuclear MSH6 staining within tumor cells. 

For a semiquantitative assessment of the level of protein expression, a total IRS score (immunoscore) was used. The protein immunoexpression data were dichotomized into negative (low expression) and positive (high expression) based on the optimal cut-point value defining with the cutp function of the Evaluate Cutpoints application, which is developed using the R language [6]. Cases defined as having high expression were those with scores equal and above the cutoff value, while low expression was associated with scores below the cutoff value. To determine the combined prognostic significance of cyclin F, RRM2, and SPDL1 proteins, the combined variables were examined by adding their respective immunoscores, and their sum was dichotomized (<10.33 or ≥10.33) for purposes of statistical analysis. 

### 2.4. Database Analysis

Gene expression data for the Cancer Genome Atlas (TCGA) and Genotype-Tissue Expression (GTEx) cohorts of patients with pancreatic adenocarcinoma were downloaded through the UCSC Xena Browser (http://xena.ucsc.edu/ (accessed on 26 August 2020)). The analyzed cohort included 177 pancreatic adenocarcinoma patients (146 of ductal histology) with median overall survival of 614 days and 165 cases of non-cancerous pancreatic tissues. The clinicopathological data were downloaded from the TCGA database using cBioPortal (http://www.cbioportal.org/ (accessed on 26 August 2020)). Grade 1, 2, 3, and 4 comprised 17.71%, 53.71%, 27.43%, and 1.14%, respectively. Stage I, II, III, and IV comprised 12%, 83.43%, 2.29%, and 2.29%, respectively. RNA-sequencing (RNA-seq) transcriptome data were normalized via DESeq2 normalization. The gene expression data were dichotomized into high level and low level groups according to cutoff points determined with the Evaluate Cutpoints software [6].

### 2.5. Statistical Analysis

Statistical analysis was carried out with the GraphPad Prism (version 7.01, GraphPad Software, La Jolla, CA, USA) and SPSS software packages (version 26.0, IBM Corporation, Chicago, IL, USA). Data normality was assessed using the Shapiro Wilk test. Continuous variables were compared using the Mann–Whitney test. The association between continuous variables was assessed by the Spearman correlation coefficient (r). Chi-squared test or Fisher’s exact test was used to compare the categorical variables. Survival outcomes were evaluated with the Kaplan–Meier method and compared via the log-rank test. Univariate and multivariate survival analyses were performed by Cox proportional hazards model to estimate the hazard ratios (HR) with 95% confidence intervals (CI). Multivariate survival analysis of our cohort of PDAC patients was done after the data were adjusted for the study (low vs. high) and established clinical parameters, including cell differentiation (well vs. moderate + poorly), age (≤60 years vs. >60 years), gender (female vs. male), AJCC pathological stage (stage I + II vs. stage III + IV), vascular invasion (absent vs. present), and perineural invasion (absent vs. present). For multivariate survival analysis of in silico data, covariates were cell differentiation (well vs. moderate + poorly + undifferentiated), T classification (T1 + T2 vs. T3 + T4), and N classification (N0 vs. N1), gender (male vs. female vs. male), and age (≤60 years vs. >60 years). Owing to the strong correlations between CCNF, RRM2, and SPDL1 mRNA expression, we did not include them in the same multivariate model. The level of significance was set at probabilities of *p* < 0.05.

## 3. Results

### 3.1. Analysis of Immunohistochemical and In Silico Gene Expression Data—Association with Clinicopathological Features and Overall Survival

Expression and subcellular distribution of the examined proteins were assessed with IHC on tissue macroarrays containing 68 PDAC specimens and 65 non-tumor adjacent tissues. Of 68 PDACs, 62 cases with both expression level of studied proteins and survival data were available for analysis. The protein expression data of our own cohort were evaluated with regard to clinicopathological features, Ki-67 index, and overall survival of PDAC patients. The analyzed data also included gene expression (mRNA-seq) data of tumor and healthy samples from the TCGA and GTEx, respectively, which were downloaded through the UCSC Xena Browser. The mRNA expression data of TCGA cohort were assessed in relation to clinicopathologic variables, MKI67 mRNA expression (coding for the Ki-67 protein), and overall survival of PAC patients. A total of 177 samples with both expression level of studied markers and survival data were available for analysis. Given that cyclin F, RRM2, and SPDL1 are considered GIN-related proteins, we also analyzed their expression in relationship to tumor aneuploidy score and fraction genome altered (TCGA cohort) or MSH6 protein level (our cohort).

#### 3.1.1. Cyclin F

**Figure 1:**
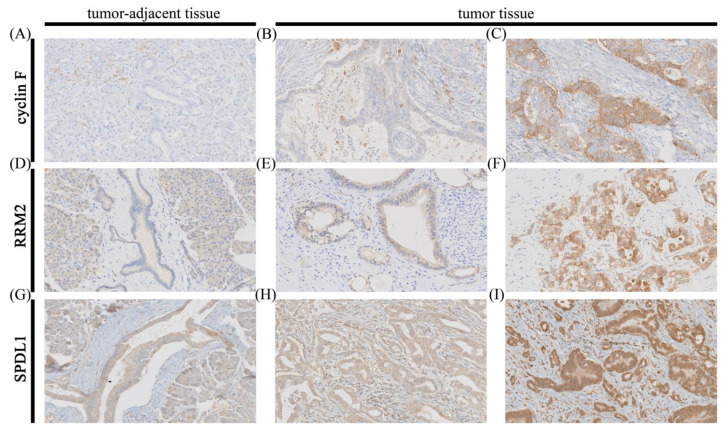
Representative images of immunohistochemical expression of cyclin F, RRM2, and SPDL1 in pancreatic ductal adenocarcinoma (PDAC) and adjacent tissue (control). Cyclin F staining in control (**A**); weak staining; (**B**) and strong staining; (**C**) for cyclin F in PDAC; RRM2 staining in control; (**D**); weak staining; (**E**) and strong staining; (**F**) for RRM2 in PDAC; SPDL1 staining in control; (**G**); weak staining; (**H**) and strong staining; (**I**) for SPDL1 in PDAC. Original magnification 20×.

Of 68 pancreatic ductal adenocarcinomas, 43 (63.24%) demonstrated cyclin F immunoreactivity, while normal pancreatic ductal epithelium was mostly non-reactive (*n* = 59/65; 90.77%). Cyclin F was focally expressed in 31 of PDAC cases, and ubiquitously expressed in 12 samples. The staining pattern was both membranous and cytoplasmic in tumor tissues and, if present, also the same in non-tumor tissues. Cyclin F labeling was granular in many PDAC cases, especially in those with strong staining intensity. Representative photomicrographs illustrating low and high IHC expression of cyclin F in PDAC and adjacent normal tissue are presented in Figure 1A–C.

**Figure 2:**
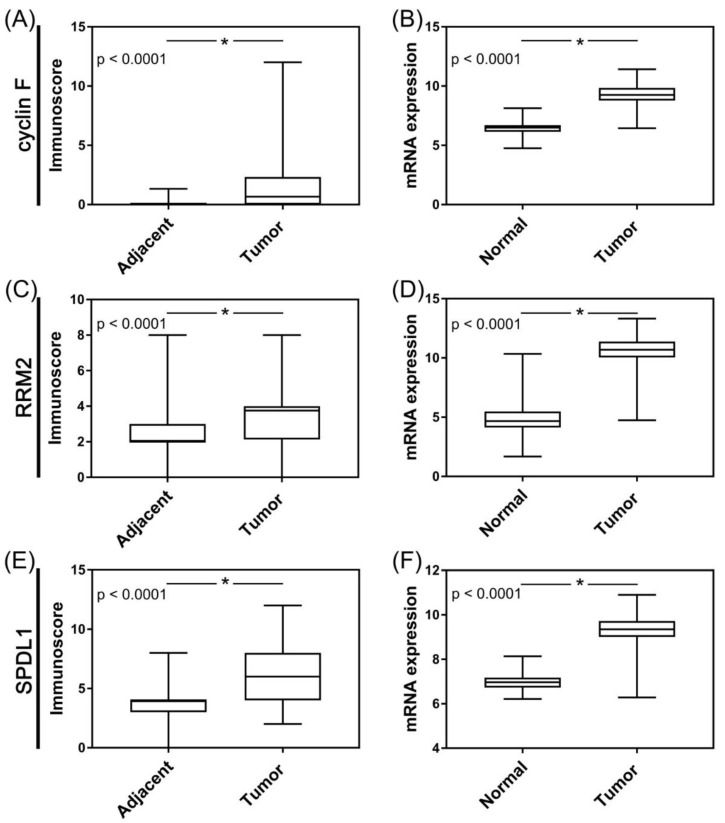
Protein and mRNA expression of cyclin F (CCNF), RRM2, and SPDL1 in pancreatic adenocarcinoma. (**A**,**C**,**E**) protein expression data were obtained by in-house immunohistochemistry; X axis of the plot represents histologically normal tissue that was adjacent to tumor tissue vs. cancer tissue, Y axis represents immunoscores (IRS). (**B**,**D**,**F**) mRNA expression data were retrieved from the Cancer Genome Atlas (TCGA); X axis of the plot represents normal vs. cancer tissue; Y axis represents normalized expression of mRNAs. The top and bottom of the error bars represent the maximum and minimum values of data, respectively. Asterisk indicates statistical significance (*p* < 0.05, Mann–Whitney test).

Cyclin F expression was obviously increased in PDAC tissues compared with non-malignant tissues adjacent to the tumor area (*p* < 0.0001; Figure 2A). Correspondingly, the TCGA dataset showed that the expression of CCNF mRNA in PAC tissues was significantly up-regulated as compared with normal pancreatic tissues (*p* < 0.0001; Figure 2B). By establishing a cut point at ≥6.67 and ≥9.17, overexpression of cyclin F was found in six (8.82%) patients of our cohort and in 102 (57.63%) patients of TCGA cohort.

Overexpression of cyclin F protein occurred significantly more frequently in PNI-negative tumors than PNI-positive tumors (22.73% vs. 2.38%; *p* = 0.02). No other relationships between cyclin F protein expression and clinicopathologic features were found (Table 1). There was also no significant correlation between cyclin F expression and Ki-67 index (r = −0.08; *p* = 0.51) or MSH6 expression (r = −0.12; *p* = 0.34). In turn, CCNF mRNA overexpression was markedly more frequent in intermediate (61.70%) and high grade (66.00%) tumors than low grade ones (32.26%; *p* = 0.01), in locally advanced (T3-T4) tumors (62.07%) than less invasive (T1-T2) tumors (40.00%; *p* = 0.04), and in stage II-IV tumors (61.04%) than stage I tumors (38.10%; *p* = 0.06). The details on this analysis are presented in Table 2. Furthermore, CCNF expression was significantly positively correlated with aneuploidy score (r = 0.32; *p* < 0.0001), fraction of genome altered (r = 0.35; *p* < 0.0001), as well as MKI67 expression (r = 0.82; *p* < 0.0001).

**Figure 3:**
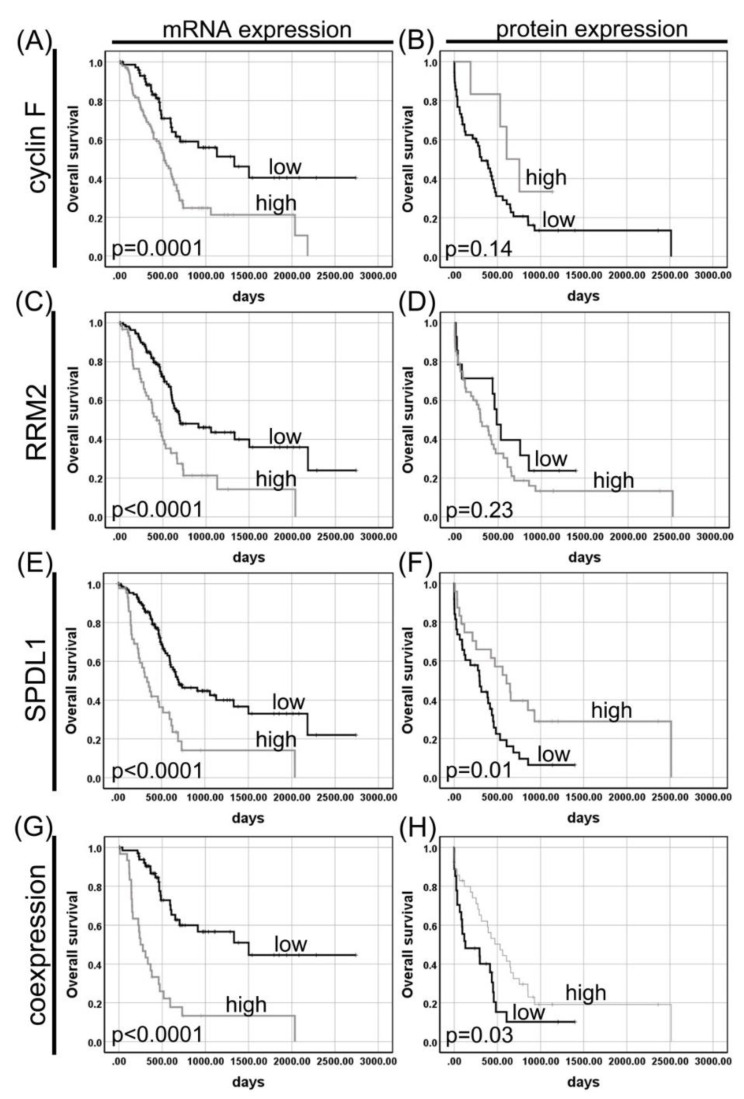
Kaplan–Meier curves for overall survival of pancreatic adenocarcinoma patients stratified by (**A**) cyclin F (CCNF) mRNA expression; (**B**) cyclin F protein expression; (**C**) RRM2 mRNA expression; (**D**) RRM2 protein expression; (**E**) SPDL1 mRNA expression; (**F**) SPDL1 protein expression; (**G**) combined CCNF/RRM2/SPDL1 mRNA expression; (**H**) combined cyclin F RRM2/SPDL1 protein expression. Protein and mRNA expression data were obtained by in-house immunohistochemistry and the Cancer Genome Atlas (TCGA) database, respectively. *p* values were calculated using the log-rank test.

Kaplan–Meier survival curves revealed a significant association between high CCNF mRNA expression and a shorter survival of patients with PAC (518 days vs. 1332 days; *p* = 0.0001; Figure 3A). In contrast, patients whose PDACs expressed high protein level of cyclin F tended to survive longer than those expressing low level of this protein (606 days vs. 314 days; *p* = 0.14; Figure 3B). 

In the univariate Cox proportional hazards model, CCNF mRNA expression was significantly associated with a poor prognosis (HR = 2.4, 95% CI 1.52–3.80; *p* = 0.0002; Table 3), while cyclin F protein expression with better prognosis, but without statistical significance (HR = 0.47, 95% CI 0.17–1.32; *p* = 0.15; Table 4). In the multivariate Cox proportional hazards model, mRNA (adjusted HR = 1.66, 95% CI 1.04–2.67; *p* = 0.03) but not protein (adjusted HR = 0.37, 95% CI 0.10–1.43; *p* = 0.15) expression of cyclin F was an independent prognostic factor for OS (Table 3 and Table 4, respectively).

#### 3.1.2. RRM2

RRM2 immunostaining was detected on 65/68 (95.59%) and 58/65 (89.23%) of the PDACs and adjacent non-neoplastic pancreatic tissues, respectively. RRM2 labeling was membranous–cytoplasmic in the vast majority of cancer cells, whereas it was mainly restricted to the cytoplasm of non-tumor cells. Cytoplasmic staining was accompanied by nuclear staining in several PDAC cases (6/68; 8.82%) and in few cells in the area of normal pancreatic tissue (2/65; 3.07%). This staining pattern had usually one degree higher intensity than the predominant pattern. Figure 1D–F presents sample IHC stains for patients with low- and high-expression profiles of RRM2 in PDAC and adjacent normal tissue.

As shown in Figure 2C, expression of RRM2 protein was significantly higher in PDAC tissues compared to normal peritumoral pancreatic tissues (*p* < 0.0001). Likewise, RRM2 mRNA was significantly elevated in pancreatic adenocarcinoma cells comparatively to their normal counterparts (*p* < 0.0001; Figure 2D). Cutoff values of 2.5 and 11.09 were used for tumor classification and patient dichotomizing into two groups according to RRM2 protein and mRNA levels, respectively. Based on these, the protein and mRNA levels of RRM2 were found to be upregulated (high expression groups) in 51 (75%) and 62 (35.03%) tumor cases of our cohort and TCGA cohort, respectively.

RRM2 protein and mRNA did not cosegregate with any clinicopathologic variables assessed (*p* > 0.05; Table 1 and Table 2, respectively). Moreover, there was no correlation between RRM2 protein level and Ki-67 index (r = 0.014; *p* = 0.91) or MSH6 expression (r = 0.09; *p* = 0.45), but the RRM2 mRNA level was significantly positively correlated with aneuploidy score (r = 0.31; *p* < 0.0001), fraction genome altered (r = 0.33; *p* < 0.0001), as well as MKI67 expression (r = 0.79; *p* < 0.0001).

Kaplan–Meier survival analysis of data from TCGA showed that PAC patients with increased RRM2 mRNA had a shorter OS. The median survival times were 430 and 695 days for high and low expression groups, respectively (*p* < 0.0001; Figure 3C). At univariate Cox analysis, poorer OS was prominently correlated with high RRM2 expression (HR = 2.39, 95% CI 1.58–3.63; *p* < 0.0001; Table 3). When examined in a multivariate analysis, high RRM2 mRNA persisted as a negative prognostic factor for OS (HR = 1.94, 95% CI 1.27–2.97; *p* = 0.002; Table 3). Conversely, high or low expression of RRM2 protein made no significant differences in predicting the overall survival of PDAC patients (*p* > 0.05; Table 4). The median survival time for the patients with high or low RRM2 expression was 297 days and 483 days, respectively (*p* = 0.23; Figure 3D).

#### 3.1.3. SPDL1 (Spindly)

A total of 68 PDAC cases were labeled immunohistochemically with the SPDL1 antibody. In tumor tissues, SPDL1 was mainly detected in the cytoplasm, but sporadic membranous–cytoplasmic staining pattern occurred, while the adjacent non-cancerous tissues showed solely cytoplasmic immunoreactivity. Representative images presenting low and high IHC expression of SPDL1 protein in PDAC and adjacent normal tissue are depicted in Figure 1G–I.

Both SPDL1 protein and mRNA levels were markedly up-regulated in tumor tissues relative to non-cancer normal tissues (*p* < 0.0001; Figure 2E,F, respectively). Overexpression of SPDL1 protein (cut point at ≥8) was detected in 28 (41.18%) PDAC cases; the remainder (40/68; 58.82%) expressed low levels of this protein. High SPDL1 mRNA levels (cut point at ≥9.72) were found in 44 (24.86%) PDAC cases, whereas low expression in the remaining 133 cases (75.14%).

The expression of SPDL1 protein tended to be elevated in PNI-negative cases compared to positive ones (*p* = 0.11). There was no other association between Spindly protein and any of the other clinicopathological traits (Table 1), or Ki-67 index (r = 0.06; *p* = 0.64). However, SPDL1 protein correlated positively with MSH6 expression (r = 0.26; *p* = 0.04). In the TCGA cohort, high mRNA level of SPDL1 was more frequently seen in T3-T4 patients than T1–T2 ones, but without reaching statistical significance (*p* = 0.11; Table 2). In addition, the expression of SPDL1 mRNA was positively correlated with aneuploidy score (r = 0.29; *p* = 0.0003), fraction genome altered (r = 0.15; *p* = 0.04), and *MKI67* expression (r = 0.59; *p* < 0.0001), but not with other features of PAC patients, such as gender, histological grade, pT, pN, or TNM stage (*p* > 0.05; Table 2).

A Kaplan–Meier survival analysis of the TCGA cohort revealed that the high SPDL1 expression group showed a significantly shorter median OS than the low expression group (695 days vs. 334 days; *p* < 0.0001; Figure 3E). In the univariate Cox proportional hazards model, *SPDL1* mRNA expression was significantly associated with a poor prognosis (HR = 2.80, 95% CI 1.81–4.31; *p* < 0.0001; Table 3). In the multivariate Cox analysis, after adjusting for clinical factors, SPDL1 remained an independent poor prognostic factor for OS (HR = 2.39, 95% CI 1.53–3.75; *p* = 0.0001; Table 3).

Contradictive to mRNA expression, a Kaplan–Meier analysis of our cohort showed that high expression of SPDL1 protein was significantly associated with better OS (294 days vs. 606 days; *p* = 0.01; Figure 3F). The favorable prognostic effect of SPDL1 expression was also observed in the univariate Cox analysis (HR = 0.45, 95% CI 0.24–0.83; *p* = 0.01; Table 4). In the multivariate analysis, the Cox proportional hazards model was applied to evaluate whether the SPDL1 protein showed an independent prognostic significance. According to this analysis, overexpression of SPDL1 protein was found to be an independent predictor of improved OS after adjustment for age at diagnosis, gender, histologic grade, tumor stage, VI and PNI, and study (HR = 0.36, 95% CI 0.16–0.80; *p* = 0.01; Table 4).

### 3.2. Coexpression of Cyclin F, RRM2, and SPDL1 in Pancreatic Adenocarcinoma and Its Impact on Patient Survival

There were highly significant correlations between mRNA levels of all examined markers in PAC tissues of the TCGA cohort. In more detail, Spearman correlation coefficient statistics revealed strong positive correlations between CCNF and RRM2 (r = 0.76; *p* < 0.0001), CCNF and SPDL1 (r = 0.60; *p* < 0.0001), as well as between SPDL1 and RRM2 (r = 0.64; *p* < 0.0001). Importantly, patients whose PAC simultaneously expressed CCNF, RRM2, and SPDL1 at high level had dramatically shorter survival time compared to those patients whose tumor tissue expressed all these markers at low level (250 days vs. 1502 days; *p* < 0.0001; Figure 3G; HR = 4.64, 95% CI 2.61–8.27; Table 5), or those patients whose PACs expressed only one of these markers at high level (*p* = 0.04). Furthermore, overexpression of the combined variable of CCNF, RRM2, and SPDL1 was the most significant independent prognostic marker for a worse OS after adjustment for age at diagnosis, gender, histologic grade, pT, and pN (HR = 3.51, 95% CI 1.93–6.36; *p* < 0.0001; Table 5).

We also compared the IRS scores between the three markers in our cohort, and none of the proteins were found to significantly correlate with one another. The Spearman correlation coefficient (r) was −0.10, −0.20, and 0.08 between cyclin F and RRM2 (*p* = 0.43), cyclin F and SPDL1 (*p* = 0.11), and SPDL1 and RRM2 (*p* = 0.54), respectively. We next examined the prognostic significance of the combined cyclin F, RRM2, and SPDL1 protein profile. Although there was a statistically significant survival difference between the two groups (129 days vs. 531 days; *p* = 0.03; Figure 3H), these three proteins in combination were not able to better predict patient survival than each of the proteins individually (*p* = 0.21).

## 4. Discussion

Although the field of oncology has recently experienced considerable changes with the cancer biomarkers and personalized cancer therapy, their clinical use in the management of PDAC patients lags behind other solid tumors. A diverse array of PDAC markers in terms of their diagnostic, predictive, and prognostic potential has been proposed, but to date, no clinical benefit of these has been realized, therefore further studies are needed. Defective cell cycle machinery, mitotic checkpoints, and DNA repair are features of genomic instability, and represent opportunities for finding novel biomarkers, as well as therapeutic targets, since many of the elements of GIN-related processes correlate with cancer cell growth, survival, and metastasis in both in vitro and in vivo preclinical models, while they are differentially expressed and/or have prognostic value for patients with various cancer types [3,7]. With the aim of a joint global effort to find novel biomarkers and therapeutic targets for this deadly tumor, in the present study, we evaluated the immunohistochemical expression of three GIN-related proteins: cyclin F, RRM2, and SPDL1 with regard to clinicopathological characteristics and survival outcomes of PDAC patients. We also compared the protein expression detected in our cohort with mRNA-seq data of PAC cases obtained from public sources.

Cyclin F is the founding member of the F-box family of proteins, which constitute the substrate recognition subunits of SCF (Skp1–Cul1–F-box protein) ubiquitin ligase complexes. This is an orphan cyclin that oscillates during the cell cycle, peaking in G2, but it does not bind or activate any cyclin-dependent kinases (CDKs). Cyclin F has been implicated in the control of centrosome replication, mitotic spindle organization, and cellular dNTP pools through targeting CP110, NuSAP1, and RRM2, respectively, for proteasome-mediated degradation. Based on these findings, its role in controlling genome integrity through ubiquitin-mediated proteolysis has been proposed [8,9,10,11,12]. Despite its crucial nature and role in mitotic fidelity, the expression of this protein with respect to its prognostic significance in human cancers has hardly been studied, and most available articles, including our recent one [13], are based on in silico transcriptome data, but to our knowledge, none of the previous ones concerned the pancreatic cancer. Here, the analysis of TCGA dataset of 177 pancreatic adenocarcinoma patients showed that mRNA expression levels of CCNF were significantly elevated in 57.63% of PDACs and associated with more advanced and aggressive tumors and poor patient survival. Specifically, multivariate Cox analysis revealed that CCNF mRNA expression was an independent predictor of worse OS after adjustment for histologic grade, tumor status, nodal status, age, and gender (HR = 1.66, 95% CI 1.04–2.67; *p* = 0.03). In agreement with our studies, CCNF has been recently identified as an upregulated gene in primary pancreatic tumors from Ela-c-myc transgenic mice, compared to both normal pancreas and liver metastatic lesions [14]. In terms of clinical significance, the results obtained here are in line with our recent non-pancreas cancer in silico study, in which overexpression of CCNF mRNA was associated with a worse survival of melanoma patients [13]. Based on publicly available datasets, Liu et al. have also shown that CCNF was positively correlated with the types of highly malignant and poor-prognostic breast cancer (BC), which had the features of low differentiation, high invasiveness, easy to metastasize, and relapse. Furthermore, they found that increased expression of CCNF mRNA was associated with worse OS, relapse free survival (RFS), distant metastasis free survival (DMFS), and post progression survival (PPS), and further demonstrated the elevated levels of cyclin F protein in BC tissues using IHC [15]. Similar studies of Wang et al. [16] have also revealed that high mRNA expression levels of CCNF denote a poor prognosis in BC patients, whereas Li et al. [17] have reported corresponding findings for hepatocellular carcinoma (HCC). Conversely, CCNF mRNA levels correlated with better survival outcome in colorectal cancer (CRC), as it has been presented by Chen et al. [18] based on the in silico analysis. In contrast to the study by Li et al. [17], Fu et al. [19] have provided evidence for high expression of cyclin F mRNA and protein being correlated with better survival of HCC patients. Here, we also observed that patients with tumors that express more cyclin F protein survived longer than those with its low expression, though our results were not statistically significant (606 days vs. 314 days; *p* = 0.14). A similar controversy as to the functional portrait of this atypical cyclin has been presented in the experimental studies. Given the characteristics of the substrates of SCF-cyclin F, the latter might be expected to act as a tumor suppressor, and most experimental studies have recently supported this thesis; however, those supporting its pro-tumorigenic activity also exist [12,20,21,22]. Although overexpression correlating with a poor prognosis fits better to the best known role of cyclins in human malignancies, it is also well documented that these and virtually all cancer regulatory proteins may manifest oncogenic features in one situation but tumor-suppressive features in another [23]. Therefore, we cannot rule out that the discordance between studies may be simply attributed to disparities in tumor types/subtypes, genetic and biological heterogeneity, and sample sets (e.g., ethnic background, sample size, uneven distribution of the clinicopathological data), whereby some of these traits may be also the reason of discordance between our in silico and IHC studies. There is also possibility that cyclin F protein and mRNA may differentially modulate the clinical outcomes of PDAC patients, as it has been previously seen for other markers and tumors [24,25]. However, we have to bear in mind that cyclin F protein expression was not significantly associated with survival in our cohort, thus limiting our hypothesis of the opposite role of cyclin F protein vs. mRNA in pancreatic adenocarcinoma. This could be because the study population may have been underpowered, as a trend towards better survival was seen in high-expressing cyclin F tumors. To further verify our assumption, cyclin F expression should be evaluated in independent, larger PDAC cohort, with protein and mRNA measurement performed simultaneously on the same cases.

RRM2 is essential for DNA synthesis and repair, as it insures the activity of ribonucleotide reductase (RR). This is the only known enzyme that catalyzes the reduction of NDPs to their corresponding deoxyribonucleoside diphosphates (dNDPs) and then to deoxynucleoside triphosphates (dNTPs)—the building blocks of DNA. Apart from RRM2, another small subunit (P53R2/RRM2B) and one large subunit (RRM1) have been identified as the components of human RR. Altered expression levels of the functional enzyme have been implicated in carcinogenesis, as it is highly acknowledged that imbalanced dNTP pools can result in genomic instability and cell-cycle progression, thereby facilitating cancer cell proliferation. As far as we know, apart from the current report, only several other studies to date have assessed the clinical value of RRM2 protein [26,27] or mRNA [28,29,30,31] in pancreatic cancer cohorts, and these studies have varied with regard to the impact of RRM2 on patient survival. In terms of protein IHC staining, Fisher et al. [26] have shown that high tumor RRM2 expression was an independent negative prognostic factor for early recurrence and decreased survival in patients with early-stage PDAC, both when analyzing the entire cohort (*n* = 95) and the subset of patients receiving adjuvant therapy (*n* = 74). Our IHC results are more consistent with the conclusions of the Xie study [27], who have shown no prognostic value of RRM2 immunoexpression in 117 patients with resectable pancreatic adenocarcinoma. In the cited study, this conclusion stayed also true for a subpopulation of 44 patients subsequently treated with adjuvant gemcitabine after tumor resection [27]. Importantly, based on the analysis of the TCGA data, we identified RRM2 mRNA expression as an independent negative prognostic factor for OS in pancreatic adenocarcinoma patients. By in silico analysis of microarray-based gene expression data, Jin et al. [32] have reported similar poor prognostic effect of RRM2 for PDAC, but with regard to disease-free survival (DFS; univariate Kaplan–Meier analysis). Correspondingly, in the cohort study by Itoi et al. [28], high transcript expression of RRM2 in 31 fine-needle aspiration biopsy specimens of advanced stage pancreatic cancer significantly correlated with a poor patient survival and resistance to gemcitabine. This was further supported by the Fujita and coworkers study [30], where 70 patients with early stage pancreatic adenocarcinoma were included, whereby 40 of them were treated with gemcitabine. In both studied groups, decreased RRM2 mRNA expression was significantly associated with increased OS and progression-free survival (PSF). On the other hand, Giovannetti et al. [29], as well as Sierzega et al. [31], failed to show any significant correlation of RRM2 mRNA with the survival times of PDAC patients. Of note, a direct comparison of mRNA expression data between our study, the Jin study, and other above cited reports is limited by the fact that they originate from different quantification methods, i.e., RNA-seq, microarray, and qPCR, respectively, the results of which are not necessarily concordant. Furthermore, these studies included cases with different stages of pancreatic adenocarcinoma. Indeed, as one of the possible explanations for the apparent disagreements, it has recently been proposed that the prognostic implications of the expression profile of RRM2 could depend on the stage of pancreatic cancer [27]. Although it still cannot be ruled out, it is worth noting that Fisher et al. [26] and Xie et al. [27] exclusively evaluated patients with early-stage disease, both using immunohistochemistry, and they received inconsistent results. The majority of the patients studied in our cohort study had stage I and II disease (77.42%), although patients with stage III and IV disease were also included (22.55%). In a subset analysis limited to patients with early-stage PDAC, we were still unable to find a significant relation between RRM2 protein expression and patient survival (Figure S1). Unfortunately, our numbers were too small for reliable statistical calculation to find evidence of the marker being prognostic in late-stage disease. In addition, the technical issues related to IHC assay with a possible impact on RRM2 assessment include antibody clones, staining patterns, scoring systems, and cutoff points, as these varied substantially between our studies and those of Xie et al. and Fisher et al. Standardization of staining and scoring protocols for RRM2 IHC along with its further testing in large cohorts that are evenly distributed in terms of tumor pathological features seem crucial to establish the clinical significance of RRM2 protein in pancreatic adenocarcinoma and other tumors as well.

Spindly is a relatively recently identified regulator of mitosis [33], which has no confirmed role in cancer. It is a kinetochore-specific adaptor for cytoplasmic dynein and plays a role in chromosome alignment and spindle assembly checkpoint (SAC) signaling. Recent studies have shown that depletion of Spindly by RNAi resulted in an extensive chromosome missegregation [34,35]. In turn, Silva and co-workers [36] have hypothesized the link between high levels of this protein, CIN, and pathogenesis of oral squamous cell carcinoma (OSCC). In the recent in silico study, SPDL1 has been identified as one of the key candidate genes differentially expressed in PDAC, whereby the upregulation of its mRNA was significantly associated with a worse OS and DFS, as well as the advanced tumor stage. In the cited article of Tian et al. [37], the bioinformatics data was further confirmed in a small cohort of six PDAC patients using qRT-PCR. This study is in partial agreement with our in silico analysis, in which overexpression of SPDL1 mRNA was independently associated with a poor OS of PAC patients (adjusted HR = 2.40, 95% CI 1.53–3.75; *p* < 0.0001); however, it was not significantly correlated with any of the established clinicopathological features (*p* > 0.05), albeit there was a trend towards its higher prevalence of in pT3–pT4 tumors than pT2 and pT3 tumors (*p* = 0.11). In contrast to our results and those of Tian et al., Kodama and coworkers have shown that reduced SPDL1 mRNA expression levels are significantly associated with shorter OS in CRC patients (in silico analysis) [38].

In the present study, contradictive to mRNA expression, it was low SPDL1 protein level that was significantly associated with a worse OS of PDAC patients. To our knowledge, the present study is the first to investigate SPDL1 protein in pancreatic cancer tissues. In more detail, we found SPDL1 to be overexpressed in 41.18% of PDAC cases, and SPDL1 levels did not significantly cosegregate with the examined clinical and pathological features, though SPDL1 overexpression tended to occur more frequently in PNI-negative cases compared to positive ones (*p* = 0.11). Most importantly, however, SPDL1 protein levels allowed to stratify PDAC patients into two different survival groups. Patients with high SPDL1 expression had a better median OS than did patients with SPDL1 underexpression (606 days vs. 294 days; *p* = 0.01), with HR calculation indicating a reduced relative risk of death from any cause of 55% (95%CI 0.24–0.83, *p* = 0.01). Furthermore, SPDL1 protein sustained its significance in terms of OS after adjusting for covariates, being the most influential prognostic factor compared with clinicopathological features in patients who underwent surgical treatment (HR = 0.36, 95% CI 0.16–0.80, *p* = 0.01). These findings suggest a potentially important, but probably opposite role for SPDL1 protein and mRNA in the clinical behavior of pancreatic adenocarcinoma, but this conclusion is limited by the fact that our protein and mRNA results came from two different study population, i.e., own cohort and TCGA cohort, respectively. These differences between mRNA and protein expression with regard to prognostic relevance imply that expression measurements based on IHC, as well as RNA-seq, cDNA arrays, or qPCR, complement each other, and there is a need for a complex molecular analysis of tumors at both protein and mRNA levels. Indeed, although proteins carry out the vast majority of cellular functions and therefore are more eligible to influence prognosis, there are currently many studies providing support for the assumption that differential mRNA expression has also biological meaning [39], which should not be interpreted as a true functional relation, but rather as a process promoting mRNA transcription and/or mRNA stability of particular genes, and being itself associated with patient prognosis [40,41]. In the context of our results, it is also important to take into account that posttranscriptional and posttranslational mechanisms are likely to influence protein expression, and elevated levels of mRNA may produce only small amounts of detectable proteins [25]. Based on these, we may conclude that SPDL1 protein is probably functionally linked to superior prognosis in PDAC, whereas the opposite is true for SPDL1 mRNA. The latter suggests that any mechanisms that are responsible for induction of SDPL1 mRNA or for a negative correlation with its protein expression are potentially involved in PDAC progression and/or therapy resistance. The mechanistic support of our findings needs to be established in experimental systems. We regard this issue as important for future research.

The association of SPDL1 protein and patient survival is in contrast to previous findings of Silva et al. in OSCC [36], who have shown by IHC that SDPL1 SPDL1 is upregulated in OSCC tissues and correlated with tumor proliferation and a poor prognosis. As far as we know, there are no other reports on the impact of SPDL1 protein on disease outcome and clinical variables of cancer patients, and thus further studies are required to determine whether the role of Spindly in human cancers is tumor-type dependent.

Due to the functional relationship between the three proteins, we next compared their coexpression levels and their impact on patient overall survival. There were strong positive correlations between mRNA levels of CCNF, RRM2, and SPDL1 in PAC tissues of the TCGA cohort. Importantly, the combined variable of CCNF, RRM2, and SPDL1 was highly discriminant for OS (*p* < 0.0001, HR = 4.64, 95% CI 2.61–8.27) and was more valuable for predicting prognosis than each marker individually (*p* = 0.04). Coexpression level of studied biomarkers was the most significant independent prognostic factor associated with a shorter survival, compared to established clinicopathological features (adjusted HR = 3.51, 95% CI 1.93–6.36; *p* < 0.0001). Unexpectedly, in our cohort, cyclin F, RRM2, and SPDL1 proteins were not significantly correlated with each other (*p* > 0.05). In addition, Kaplan–Meier survival analysis failed to demonstrate that the combined expression of these proteins better predicted survival than looking at each protein individually (*p* = 0.21).

## 5. Conclusions

Collectively, the present study is the first to investigate cyclin F and SPDL1 proteins in pancreatic adenocarcinoma, and one of the few that assessed RRM2 in this group of cancer patients. Here, we identified a three-marker panel of genomic instability-related genes, whose mRNA expression levels were an independent prognostic factor for postoperative survival among PAC patients. Each of the markers alone were also highly discriminant for OS, both in the univariate and multivariate analyses. Importantly, high levels of SPDL1 protein emerged as the most powerful independent prognostic factor associated with a better outcome, compared to established clinicopathological parameters. In addition, overexpression of cyclin F protein was associated with a trend towards better survival of PDAC patients. Clinically, the two proteins would represent poor therapeutic targets, as it is the higher levels of these proteins that are correlated with better survival. However, if these data are validated in independent, larger cohorts, evaluation of SPDL1 protein as well as CCNF, RRM2, and SPDL1 mRNAs may add significant prognostic value to the currently established clinicopathologic variables and hence have potential clinical utility in the management of this deadly disease.

## Figures and Tables

**Table 1:** Association of cyclin F, RRM2, SPDL1, and the clinicopathological features in our cohort of PDAC patients (*n* = 68).

Variables	*n* (%)	Cyclin F Expression		*p*	RRM2 Expression		*p*	SPDL1 Expression		*p*
		Low *n* = 62	High *n* = 6		Low *n* = 17	High *n* = 51		Low *n* = 40	High *n* = 28	
Age (years)										
≤60	29 (42.65)	28 (96.55)	1 (3.45)	0.23	7 (24.14)	22 (75.86)	>0.99	19 (65.52)	10 (34.48)	0.31
>60	39 (57.35)	34 (87.18)	5 (12.82)		10 (25.64)	29 (74.36)		18 (50.00)	18 (50.00)	
Gender										
Male	34 (50.00)	30 (88.24)	4 (11.76)	0.67	7 (20.59)	27 (79.41)	0.58	20 (55.82)	14 (44.18)	>0.99
Female	34 (50.00)	32 (94.12)	2 (5.88)		10 (29.41)	24 (70.59)		20 (55.82)	14 (44.18)	
Grading										
G1	5 (7.35)	4 (80.00)	1 (20.00)	0.80	1 (20.00)	4 (80.00)	0.67	2 (40.00)	3 (60.00)	0.45
G2	55 (80.88)	51 (92.73)	4 (7.27)		13 (23.64)	42 (76.36)		32 (58.18)	23 (41.82)	
G3	8 (11.77)	7 (87.50)	1 (12.50)		3 (37.50)	5 (62.50)		6 (75.00)	2 (25.00)	
pT status										
T1-T2	53 (84.13)	50 (94.34)	3 (5.66)	0.51	13 (24.53)	40 (75.47)	0.71	34 (64.15)	19 (35.85)	0.18
T3-T4	10 (15.87)	9 (90.00)	1 (10.00)		3 (30.00)	7 (70.00)		4 (40.00)	6 (60.00)	
pN status										
N0	30 (45.46)	28 (93.33)	2 (6.67)	0.68	8 (26.67)	22 (73.33)	>0.99	15 (50.00)	15 (50.00)	0.21
N1-N2	36 (54.54)	32 (88.89)	4 (11.11)		9 (25.00)	27 (75.00)		24 (66.67)	12 (33.33)	
TNM stage										
I	24 (38.71)	23 (95.83)	1 (4.17)	0.56	5 (20.83)	19 (79.17)	0.56	12 (50.00)	12 (50.00)	0.46
II	24 (38.71)	21 (87.50)	3 (12.50)		8 (33.33)	16 (66.67)		16 (66.67)	8 (33.33)	
III-IV	14 (22.58)	13 (92.86)	1 (7.14)		3 (21.43)	11 (78.57)		9 (64.29)	5 (35.71)	
Location										
head	60 (88.24)	55 (91.67)	5 (8.33)	0.54	15 (25.00)	45 (75.00)	>0.99	35 (58.33)	25 (41.67)	>0.99
Body-tail	8 (11.76)	7 (87.50)	1 (12.50)		2 (25.00)	6 (75.00)		5 (62.50)	3 (37.50)	
VI										
Absent	39 (72.22)	33 (84.62)	6 (15.38)	0.17	13 (33.33)	26 (66.67)	0.51	18 (46.15)	21 (53.85)	0.23
Present	15 (27.78)	15 (100.0)	0 (0.0)		3 (20.00)	12 (80.00)		10 (66.67)	5 (33.33)	
**PNI** **Absent** **Present**	22 (34.38)	17 (77.27)	5 (22.73)	**0.02**	6 (27.27)	16 (72.73)	>0.99	10 (45.45)	12 (54.55)	0.11
	42 (65.62)	41 (97.62)	1 (2.38)		11 (26.19)	31 (73.81)		28 (66.67)	14 (33.33)	

Abbreviation: PDAC—pancreatic ductal adenocarcinoma; VI—vascular invasion; PNI—perineural invasion. Significant *p*-values (*p* < 0.05) are indicated in bold.

**Table 2:** Association of CCNF, RRM2, and SPDL1 and the clinicopathological features in TCGA cohort of PAC patients (*n* = 177).

Variables	*n* (%)	CCNF Expression		*p*	RRM2 Expression		*p*	SPDL1 Expression		*p*
		Low *n* = 75	High *n* = 102		Low *n* = 115	High *n* = 62		Low *n* = 133	High *n* = 44	
**Gender**										
Male	97 (54.80)	44 (45.36)	53 (54.64)	0.45	67 (69.07)	30 (30.93)	0.27	73 (75.26)	24 (24.74)	>0.99
Female	80 (45.20)	31 (38.75)	49 (61.25)		48 (60.00)	32 (40.00)		60 (75.00)	20 (25.00)	
Age										
≤60	59 (33.33)	26 (44.07)	33 (55.93)	0.75	39 (66.10)	20 (33.90)	0.87	44 (74.58)	15 (25.42)	>0.99
>60	118 (66.67)	49 (41.53)	69 (58.47)		76 (64.41)	42 (35.59)		89 (75.42)	29 (24.58)	
Grading										
G1	31 (17.71)	21 (67.74)	10 (32.26)	**0.01**	24 (77.42)	7 (22.58)	0.26	27 (87.10)	4 (12.90)	0.25
G2	94 (53.71)	36 (38.30)	58 (61.70)		60 (63.83)	34 (36.17)		69 (73.40)	25 (26.60)	
G3-G4	50 (28.57)	17 (34.00)	33 (66.00)		30 (60.00)	20 (40.00)		36 (72.00)	14 (28.00)	
pT status										
T1-T2	30 (17.14)	18 (60.00)	12 (40.00)	**0.04**	22 (73.33)	8 (26.67)	0.30	26 (86.67)	4 (13.33)	0.11
T3-T4	145 (82.86)	55 (37.93)	90 (62.07)		91 (62.76)	54 (37.24)		105 (72.41)	40 (27.59)	
pN status										
N0	49 (28.49)	24 (48.98)	25 (51.02)	0.24	34 (69.39)	15 (30.61)	0.48	39 (79.59)	10 (20.41)	0.44
N1	123 (71.51)	48 (39.02)	75 (60.98)		77 (62.60)	46 (37.40)		90 (73.17)	33 (26.83)	
TNM stage										
I	21 (12)	13 (61.90)	8 (38.10)	0.06	15 (71.43)	6 (28.57)	0.63	18 (85.71)	3 (14.29)	0.29
II-IV	154 (88)	60 (38.96)	94 (61.04)		98 (63.64)	56 (36.36)		113 (73.38)	41 (26.62)	

Abbreviation: PAC—pancreatic adenocarcinoma; TCGA—the Cancer Genome Atlas. Significant *p*-values (*p* < 0.05) are indicated in bold.

**Table 3:** Univariate and multivariate Cox proportional hazards models for OS of TCGA patients with PAC (*n* = 177).

Variable	Univariate Analysis				Multivariate Analysis: CCNF				Multivariate Analysis: RRM2				Multivariate Analysis: SPDL1			
	HR	95% CI		*p*	HR	95% CI		*p*	HR	95% CI		*p*	HR	95% CI		*p*
		Lower	Upper			Lower	Upper			Lower	Upper			Lower	Upper	
*CCNF*	2.40	1.52	3.80	**0.0002**	1.66	1.04	2.67	**0.03**	-	-	-	-	-	-	-	-
*RRM2*	2.39	1.58	3.63	**<0.0001**	-	-	-	-	1.94	1.27	2.97	**0.002**	-	-	-	-
*SPDL1*	2.80	1.81	4.31	**<0.0001**	-	-	-	-	-	-	-	-	2.39	1.53	3.75	**0.0001**
age	1.41	0.90	2.21	0.13	1.27	0.80	2.03	0.32	1.24	0.78	1.99	0.36	1.24	0.78	1.98	0.37
gender	0.81	0.54	1.23	0.33	0.86	0.56	1.32	0.48	0.86	0.56	1.32	0.50	0.79	0.52	1.21	0.28
grade	2.18	1.15	4.13	**0.02**	1.49	0.77	2.89	0.24	1.69	0.88	3.24	0.12	1.90	0.98	3.69	0.06
pN	2.10	1.25	3.52	**0.005**	1.90	1.09	3.30	**0.02**	1.86	1.07	3.25	**0.03**	1.93	1.11	3.38	**0.02**
pT	2.21	1.14	4.28	**0.02**	1.37	0.67	2.81	0.39	1.44	0.70	2.97	0.32	1.20	0.58	2.50	0.62
stage	0.74	0.23	2.34	0.60	-	-	-	-	-	-	-	-	-	-	-	

Abbreviations: HR—hazard ratio; CI—confidence interval; OS—overall survival; PAC—pancreatic adenocarcinoma; TCGA—the Cancer Genome Atlas. *p*-values adjusted for age, gender, histologic grade, pN, pT, and each marker separately. “-“ indicates variable was not included in multivariate analysis. Significant *p*-values (*p* < 0.05) are indicated in bold.

**Table 4:** Univariate and multivariate Cox proportional hazards models for OS of PDAC patients (*n* = 62).

Variable	Univariate Analysis				Multivariate Analysis			
	HR	95% CI		*p*	HR	95% CI		*p*
		Lower	Upper			Lower	Upper	
Cyclin F	0.47	0.17	1.32	0.15	0.37	0.10	1.43	0.15
RRM2	1.52	0.76	3.06	0.24	1.07	0.45	2.56	0.87
SPDL1	0.45	0.24	0.83	**0.01**	0.36	0.16	0.80	**0.01**
age	1.38	0.76	2.48	0.29	2.30	1.06	5.02	**0.04**
gender	1.06	0.60	1.85	0.85	1.12	0.52	2.40	0.78
grade	1.43	0.44	4.61	0.55	1.11	0.20	6.07	0.91
pN	1.07	0.60	1.91	0.81	-	-	-	-
pT	0.91	0.41	2.03	0.81	-	-	-	-
stage	1.87	0.96	3.65	0.07	2.44	1.06	5.62	**0.04**
PNI	1.80	0.96	3.35	0.07	1.40	0.61	3.20	0.43
VI	2.44	1.24	4.81	**0.01**	2.32	0.97	5.54	0.06

Abbreviations: HR—hazard ratio; CI—confidence interval; OS—overall survival; PDAC—pancreatic ductal adenocarcinoma; PNI—perineural invasion; VI—vascular invasion *p*-values adjusted for age, gender, histologic grade, tumor stage, PNI, VI, and study. “-“ indicates variable was not included in multivariate analysis. Significant *p*-values (*p* < 0.05) are indicated in bold.

**Table 5:** Univariate and multivariate Cox proportional hazards models for OS of TCGA patients with PAC (*n* = 99).

Variable	Univariate Analysis				Multivariate Analysis			
	HR	95% CI		*p*	HR	95% CI		*p*
		Lower	Upper			Lower	Upper	
CCNF + RRM2 + SPDL1	4.64	2.61	8.27	**<0.0001**	3.51	1.93	6.36	**<0.0001**
age	1.62	0.87	3.03	0.13	1.34	0.70	2.55	0.37
gender	0.94	0.53	1.66	0.83	1.22	0.67	2.23	0.51
grade	3.66	1.51	8.86	**0.004**	2.89	1.14	7.34	**0.03**
pN	2.74	1.28	5.91	**0.01**	2.69	1.05	6.91	**0.04**
pT	3.20	1.25	8.18	**0.02**	1.26	0.38	4.16	0.70

Abbreviations: HR, hazard ratio; CI, confidence interval; OS–overall survival; PAC–pancreatic adenocarcinoma; TCGA–the Cancer Genome Atlas p-values adjusted for age, gender, histologic grade, pN, pT, and coexpressionof studied markers. Significant *p*-values (*p* < 0.05) are indicated in bold.

## Data Availability

Publicly available datasets were analyzed in this study. This data can be found here: http://www.cbioportal.org/study/summary?id=paad_tcga_pan_can_atlas_2018 (accessed on 26 August 2020); https://xenabrowser.net (accessed on 26 August 2020). The own data presented in this study are available on request from the corresponding author. The data are not publicly available due to ethical restrictions.

## Supplementary Materials

The following are available online at https://www.mdpi.com/2072-6694/13/4/859/s1: Table S1: Clinicopathological properties of 68 patients with PDAC, Table S2: Antibodies and staining conditions used for immunohistochemistry, Figure S1: Kaplan–Meier curve for overall survival in patients with TNM stage I and II pancreatic ductal adenocarcinomas by RRM2 protein expression.
