# Supplementary material for: Expression of Genomic Instability-Related Molecules: Cyclin F, RRM2 and SPDL1 and Their Prognostic Significance in Pancreatic Adenocarcinoma
Source: Cancers (Basel). 2021 Feb 18;13(4):859. doi: 10.3390/cancers13040859 (PMC7922901; doi:10.3390/cancers13040859)
Supplement: Supplementary file 1 [file cancers-13-00859-s001.pdf]

Supplementary data for the manuscript:

Expression of genomic instability-related molecules: cyclin F, RRM2 and SPDL1 and their prognostic significance in pancreatic adenocarcinoma.

Anna Klimaszewska-Wiśniewska[*1], Karolina Buchholz[*1,2], Izabela Neska-Długosz[1], Justyna Durślewicz[1], Dariusz Grzanka[1], Jan Zabrzyński[3], Paulina Sopońska[4], Alina Grzanka[2], Maciej Gagat[2]

Suppl. Table 1. Clinicopathological properties of 68 patients with PDAC.

Feature	n (%)	No. of cases with data availability
**Gender**		68
Male	34 (50.00)	
Female	34 (50.00)	
**Age**		68
≤ 60	29 (42.65)	
> 60	39 (57.35)	
**Tumor grade**		68
G1	5 (7.35)	
G2	55 (80.88)	
G3	8 (11.77)	
**pT status**		63
T1	10 (15.87)	
T2	43 (68.25)	
T3	8 (12.70)	
T4	2 (3.18)	
**pN status**		66
N0	30 (45.46)	66
N1	25 (37.88)	
N2	11 (16.67)	
**cM**		68
Present	2 (2.94)	
Absent	66 (97.10)	
**Tumor location**		68
head	60 (88.24)	
body	5 (7.35)	
tail	3 (4.41)	
**VI**		54
Present	15 (27.78)	
Absent	39 (72.22)	
**PNI**		64

Present	42 (65.63)	
Absent	22 (34.37)	
**TNM stage**		62
IA	6 (9.68)	
IB	18 (29.03)	
IIA	4 (6.45)	
IIB	20 (32.26)	
III	12 (19.35)	
IV	2 (3.23)	
**Survival status**		62
alive	12 (19.35)	
deceased	50 (80.65)	

PDAC – pancreatic ductal adenocarcinoma; VI – vascular invasion; PNI – perineural invasion

Suppl. Table 2. Antibodies and staining conditions used for immunohistochemistry.

Antibody	Company	Antigen retrieval	Incubation time/dilution	Detection kit	Apparatus	Substrate
Cyclin F (PA5-67108)	ThermoFisher Scientific	high pH CC1 buffer; 64 min	40 min/1:100	Ventana ultraView Universal DAB Detection Kit	*BenchMark ULTRA*	DAB
RRM2 (PA527856)	ThermoFisher Scientific	high pH buffer, 20 min	30 min/1:1500	*Dako Envision Flex Kit*	Dako Autostainer	DAB
SPDL1 (PA560797)	ThermoFisher Scientific	high pH CC1 buffer, 64 min	32 min/1:100	Ventana ultraView Universal DAB Detection Kit	*BenchMark ULTRA*	DAB
Ki-67 (30-9)	Ventana/Roche	high pH CC1 buffer, 36 min	4 min/RTU	Ventana ultraView Universal DAB Detection Kit	*BenchMark ULTRA*	DAB
MSH6 (44)	Ventana/Roche	high pH CC1 buffer, 64 min	16 min/RTU	Ventana OptiView Universal DAB Detection Kit	*BenchMark ULTRA*	DAB

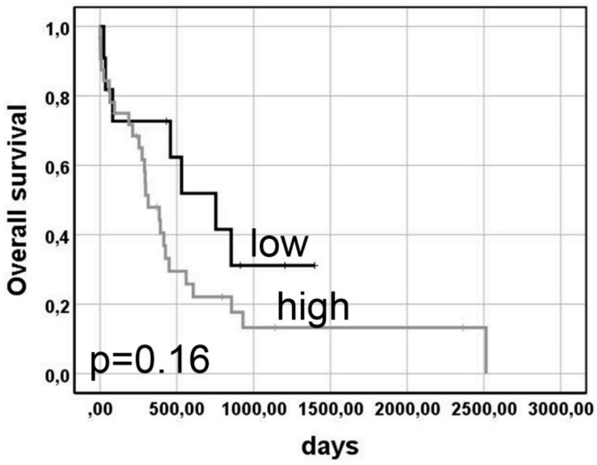

Supplementary Figure S1. Kaplan-Meier curve for overall survival in patients with TNM stage I and II pancreatic ductal adenocarcinomas by RRM2 protein expression. P value was calculated using the log-rank test.
